# Europe PMC: a full-text literature database for the life sciences and platform for innovation

**DOI:** 10.1093/nar/gku1061

**Published:** 2014-11-06

**Authors:** The Europe PMC Consortium

**Affiliations:** 1 European Molecular Biology Laboratory, European Bioinformatics Institute (EMBL-EBI), Wellcome Trust Genome Campus, Hinxton, Cambridge CB10 1SD, UK; 2 The British Library, 96 Euston Road, London NW1 2DB, UK; 3 Mimas, Roscoe Building, The University of Manchester, Oxford Road, Manchester M13 9PL, UK; 4 National Centre for Text Mining, School of Computer Science, University of Manchester, 131 Princess Street, Manchester M1 7DN, UK

## Abstract

This article describes recent developments of Europe PMC (http://europepmc.org), the leading database for life science literature. Formerly known as UKPMC, the service was rebranded in November 2012 as Europe PMC to reflect the scope of the funding agencies that support it. Several new developments have enriched Europe PMC considerably since then. Europe PMC now offers RESTful web services to access both articles and grants, powerful search tools such as citation-count sort order and data citation features, a service to add publications to your ORCID, a variety of export formats, and an External Links service that enables any related resource to be linked from Europe PMC content.

## BACKGROUND

The core mission of Europe PMC (http://europepmc.org) is to build open, full-text scientific literature resources and support innovation by engaging users, enabling contributors and integrating related research data.

Europe PMC does this primarily by providing access and adding value to over 29 million abstracts and 3 million full-text articles. It was first described in the 2011 NAR database issue (1) when it was operating as UKPMC; however in November 2012 it was rebranded as Europe PMC to reflect the Europe-wide funders that support it. Europe PMC is now the designated repository for the open access publication mandates of 26 funders of life sciences, including international organizations such as the European Research Council and World Health Organization. Europe PMC is a partner in PMC International (PMCi), along with PMC USA and PMC Canada (http://www.ncbi.nlm.nih.gov/pmc/about/pmci/), which means that full-text content is shared by and available from any of these three nodes. The main Europe PMC database is hosted at EMBL-EBI, where this core full-text collection is extended with all PubMed abstracts, selected Agricola abstracts (http://www.ntis.gov/products/agricola.aspx), international patents from the European Patent Office and abstracts from several other smaller sources.

To be clear, as with PubMed or PMC (2), the content of Europe PMC includes abstracts and articles from anywhere in the world, and they are not limited by funder or geographical location. The full scope of content in PubMed and PMC is available from Europe PMC and delivered through a single search interface.

Where Europe PMC differs from PMC/PubMed is in the services and features layered on top of the content. Since 2011, we have added several new search features and tools, which are described below.

## EUROPE PMC CORE CONTENT

It is important to note that Europe PMC contains both abstracts and full-text articles in a single search interface.

### Full-text articles in Europe PMC: correspondence with PMC

There are four ways that full-text content is added to Europe PMC and PMC, which were outlined in the previous NAR article (1) (see Box 1). If you want your article to be archived in Europe PMC, and the work is funded by one of the Europe PMC funders, then all of those four options are open to you. However if the work is not funded by one of the Europe PMC funders, then the simplest way to ensure your article is archived in PMCi is to publish your work in a journal that deposits 100% of its content into the archive. Paying an article processing charge (APC) and publishing your article in the Open Access track of a ‘hybrid’ journal does not always result in the article being deposited in the PMCi archives—this can also depend on the funder—so if you are paying an APC and expect your article to therefore become available in Europe PMC, it is worth checking that this is indeed the case. To self-archive manuscripts in Europe PMC via Europe PMC plus, you must be funded by one of the Europe PMC funders.

Box 1. **The four routes of article deposition into Europe PMC**

1. The journal makes 100% of its content available in the archive
2. The journal deposits content on an article-by-article basis
3. The final, accepted manuscript of an article is deposited via the Manuscript Submission System
4. The article is available as a part of the PMC Back Issue Digitization Project.

For more details on these deposition routes, see Ref (1).

### Abstracts in Europe PMC: correspondence with PubMed

**Figure 1.**
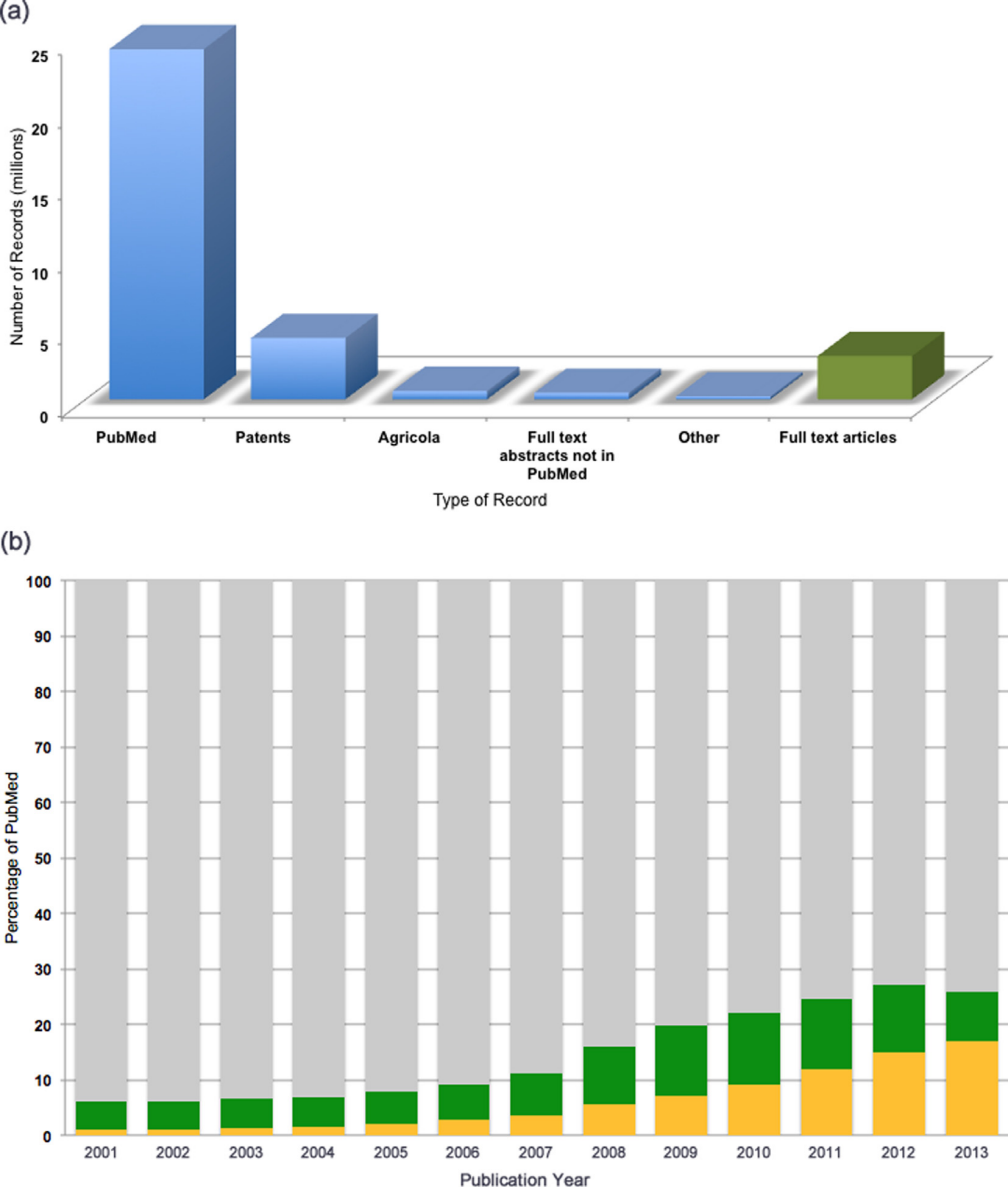
(**a**) Total scope of content (abstracts and articles) available in Europe PMC. Blue columns represent abstracts; the green column represents full text. Actual numbers of records at the time of writing: PubMed: 24 174 318; Patents: 4 229 297; Agricola: 577 871; Europe PMC full-text abstract not in PubMed: 447 427; Other smaller sources of metadata: 201 988; Europe PMC full text: 2 988 235. (**b**) Proportion of full-text articles in Europe PMC expressed, for comparative purposes, as a proportion of abstracts available in PubMed (100%, gray bar), per publication year. Open Access full-text articles are in yellow; ‘read only’ full-text articles are in green. For publication year 2012, around 25% of PubMed is available as full text in Europe PMC, and of those, about 50% are available open access. The slight drop in read-only articles seen in 2013 is due to many articles published in 2013 still being under a 12-month embargo period at the time of writing.

Europe PMC supplements the full-text article collection with abstracts from a variety of sources. In particular, Europe PMC ingests all of PubMed, updated daily (currently about 24 million records), adds records from the agricultural collection Agricola monthly (about 600 000 records) and contains international patent abstracts from the life sciences domain and ad hoc additions of metadata from other smaller sources. In cases where a full-text article in Europe PMC does not have a PubMed record, we also create a metadata record—there are about 440K of these, and they reflect records in flux (not yet in PubMed but will be) or records for articles of a type not taken by PubMed. The total content available in Europe PMC at the time of writing can be seen in Figure 1a.

### Open access: why licensing is important

While all of the content in Europe PMC is free to read, not all of it is free to reuse, for example to redistribute, translate or text mine. At the time of writing, about 870 000 articles are open access, i.e. they can be reused based on criteria stipulated in the article license, and this number increases daily. These articles are available for bulk download on the Europe PMC FTP site (http://europepmc.org/FtpSite), and are also served in full through the Europe PMC web services (see below). The open access article set makes up a growing proportion of the total full-text content available via Europe PMC (Figure 1b).

## SEARCHING EUROPE PMC IS EASY AND POWERFUL

Several improvements have recently been made to the Europe PMC search service. Here we describe a few highlights. The full list of fields available to search can be found in Europe PMC Help. While this section describes how to use these features via the website, all are also enabled in the RESTful web services.

### Citation count sort order

The articles can now be returned ranked by the number of citations. For example, it is now easy to browse classic papers (i.e. the most highly cited articles) in Europe PMC.

### Author search

If you search Europe PMC for an author name, for example Bradford MM or Janet Thornton, we automatically detect that this looks like an author name and limit the search to the author field. This results in a cleaner results list than would have been retrieved previously. If this default behavior proves problematic, you can force a different behavior via the new Advanced Search form.

### Advanced search

Use this form (found under the wheel icon to the right of the search box) if you know specifically what you are looking for. Many fields operate with autosuggest—which is particularly useful for restricting searches to a journal name, for example. The Advanced Search also makes it easy to find records that cite data (3) or are linked from various data resources; for example, articles that cite PDB, articles cited in Uniprot or abstracts that have freely available full text in institutional repositories that are not available in Europe PMC (under the External Links tab on abstract pages).

### Section search

One of the new options on the Advanced Search relates only to the full-text article set: Section Search (submitted, J. Biomed. Semantics). Here, it is possible to limit searches to specific sections such as Acknowledgments (try searching for papers that have acknowledged you) or figure legends.

Finally, when you find something that you want to share, there is a tweet button on every article page.

## EUROPE PMC AND ORCID

### Link publications to your ORCID

ORCIDs are unique identifiers for researchers, allowing them to unequivocally showcase their work and make publication processes more straightforward (http://orcid.org). We have developed a simple tool for researchers to link publications found in Europe PMC to their ORCID record (see: http://europepmc.org/orcid/import). So far, about 17 000 unique ORCIDs have been linked to over 300 000 publication claims using this tool. The tool is available from the Europe PMC homepage, and is also discoverable on the ORCID Foundation website.

### Use ORCID to search Europe PMC

**Figure 2.**
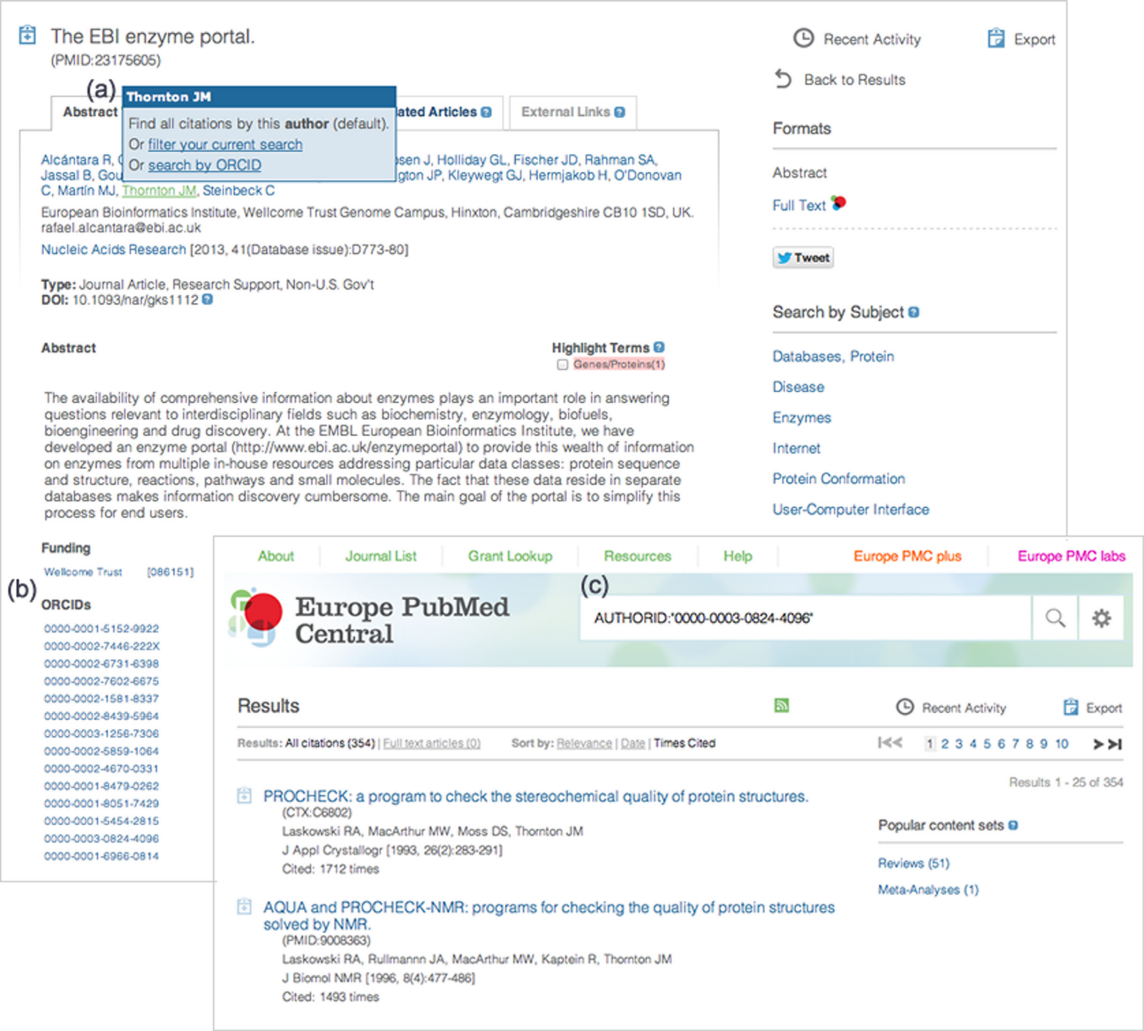
ORCIDs in Europe PMC. ORCIDs can be used to search for authors unambiguously in Europe PMC, and are apparent in several different ways. Abstract pages: (**a**) If the ORCID has been matched to a specific author in an article, there is an option to use the ORCID instead of the name to search for a particular author. (**b**) ORCIDs that have claimed the article are listed below the abstract; the name linked to the ORCID is shown on mouse-over and clicking on the ORCID triggers a search. Search page: (**c**) If you know an ORCID, search for it using the syntax shown, or select ‘ORCID’ from the Bibliographic Fields menu on the Advanced Search Page. Here the results are shown ranked by citation count.

Europe PMC has integrated ORCIDs into article records and search systems (Figure 2). If someone has claimed an article, the ORCID is displayed below the abstract; clicking on it will elicit a search based on that ORCID. A search by ORCID is also available as an option by clicking on an author name. If you know someone's ORCID, the correct search syntax can be ensured by selecting ‘ORCID’ from the pull-down list of Bibliographic Fields in the Advanced Search page. This is a particularly useful approach for people who have frequently occurring last names, or have changed their name.

## INTEGRATING DATA WITH LITERATURE VIA EUROPE PMC

The idea that in the future, the scientific article stands shoulder-to-shoulder with data, seamlessly integrated and enriched with structured information, is a vision that has emerged over the past decade (3), and is something that Europe PMC actively contributes to. To this end, we have recently developed new ways to foster links between the literature and data:

### Database records that cite articles

In addition to the database links described in the 2011 article, these now also include OMIM (4) and ArrayExpress (5), and are updated at least monthly. Several large central databases such as UniProt (6) are covered (to date over 900 000 articles are associated with UniProt) as well as thematic databases such as the Genome RNAi database (7). The External Links service is open to any database that cites the life science literature and uses a simple combination of Excel spreadsheet (or XML) and FTP to upload lists of article-database links, which are then published to the website, usually within 24 h. The External Links service can also be used by repositories that hold the full-text articles for which Europe PMC only has the metadata. See: http://europepmc.org/LabsLink for more details.

### Articles that cite data

Europe PMC now routinely discovers several types of data citations in the form of accession numbers, for example to the European Nucleotide Archive (8), and DOIs out of the full text of Europe PMC articles (9) via the Europe PMC text-mining engine, which is based on Whatizit (10). Since 2013, these and Europe PMC's article citation counts have been incorporated into the PLOS Article Level Metrics system.

### Text-mined concepts

As well as text mining gene/protein names, organisms, chemicals, diseases, and Gene Ontology terms (11), Experimental Factor Ontology (EFO) terms are now also routinely mined as a force for data integration (12). (The EFO is an ‘umbrella’ ontology designed to work across a wide range of biological domains).

**Figure 3.**
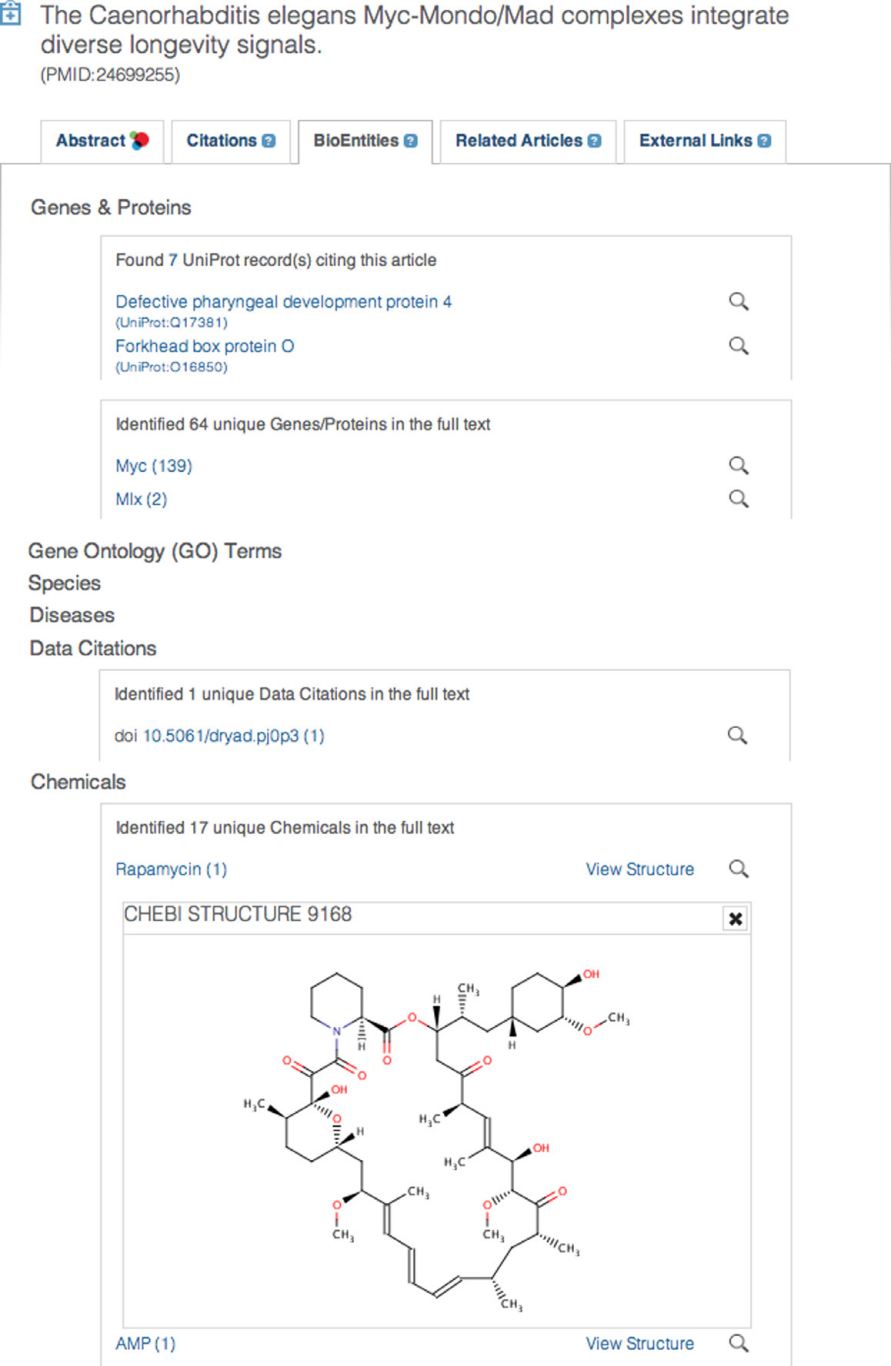
The BioEntities tab in Europe PMC. The BioEntities tab contains rich information on key concepts mined from the article as well as links to and from related data resources. In this example, several Uniprot records cite this article, and we have also found one data citation, to a dataset in the Dryad database (http://datadryad.org). In the case of chemical entities mined from text, we have used a BioJS module (http://www.ebi.ac.uk/Tools/biojs/registry/) to display the chemical structure of the entity (13). The same applies to mined PDB citations (not shown). In other cases, links to the database in question, or to invoke a search of Europe PMC for that concept, are provided. This figure has been modified and abbreviated from the actual screenshot to show the breadth of coverage in this example article.

All the data-literature citations and crosslinks can be explored through the BioEntities and External Links tabs on abstract and article pages (Figure 3), ‘Data Links and Citations’ and ‘External Links’ menus on the Advanced Search page, and through the Europe PMC APIs.

## DOWNLOADING DATA FROM EUROPE PMC

Europe PMC offers several routes to download data, depending on what you want to do and the scale at which you want to do it.

### Export articles from the website

For occasional use (for example, downloading a bibliography, inserting a citation into a document or checking the full-text XML of an Open Access article), select records from a search results list, or, to download the whole list (or single article), just click on ‘Export’ to the right of the page. The download formats offered are:

- ID list (PMIDs and others)
- BibTex
- RIS (for use to export to a variety of citation manager products)
- Tab separated values
- Citation (core citation details in plain text form for inserting into documents)
- XML (metadata including abstracts)
- Full-text XML (Open Access articles only)

There is no limit on the number of records that can be downloaded via this route, although if the number is more than 2000, then it has to be done in batches. We strongly recommend that you use the APIs described below if you are gathering more than a few hundred records, or if you are retrieving information routinely

### Web services and APIs

#### Articles

Europe PMC offers a modular RESTful API that outputs in XML or JSON formats, through which it is possible to search all content and retrieve metadata, plus full text, figures and supplementary files for the Open Access full-text articles. In the case of full-text articles that do not have a license that allows redistribution, the full text is searched but only metadata will be returned. A Dublin Core output format is also available for metadata.

#### Grants

The 26 funders of Europe PMC supply grant award data to Europe PMC. These data are used to validate researchers funded by any of the Europe PMC funders so that those researchers can use the Europe PMC manuscript submission system, Europe PMC plus. However, all the public grant data submitted by the funders can also be searched and browsed in the Grant Lookup Tool, and, more recently, are also available via a public RESTful API.

### FTP and RSS

For bulk download of the XML of the Open Access article set, visit the Europe PMC FTP site. The main article set is updated weekly. A quarterly snapshot is archived to make it easier to cite the collection as dataset.

Finally, if you like information to be pushed, you can set up an RSS feed for your favorite search. This could include, for example, articles that cite a particular data type (e.g. protein structures) or cite a particular article, or articles by a specific author, institute or on a topic of interest.

## EUROPE PMC RESOURCES FOR TEXT AND DATA MINERS

The Europe PMC core text mining service provides some basic utility to integrate literature and data. However, we envision that algorithms and applications based on text and data mining, developed by the research community, will add significant value to the content, in particular to the growing Open Access article set in the context of biological data. To this end, Europe PMC aspires to provide text and data miners with the means to publish the outcomes of their algorithms in the context of articles in Europe PMC. This is exemplified by the Evidence Finder application, which is invoked on the full-text search for queries based on gene/protein names, diseases, organisms, chemicals and anatomical entities (14), extracted using the text mining workbench Argo (15,16). Evidence Finder shows facts or claims, together with their interpretations (17), hidden in full-text articles based on the user's query, displaying relevant text fragments alongside the article metadata on search results lists on Europe PMC Labs. For each query, it also displays a list of automatically generated questions (e.g. ‘*What causes melanoma*?’) that can be used by the user to narrow down the search results to only those documents that contain the answer to a selected question.

The External Links service provides a straightforward means to publish the outputs of text mining. In this way, phrases or triples identified by the text mining of full text can be linked to other articles, data or websites that provide useful extra information related to the source article. More information on the External Links service can be found here: http://europepmc.org/LabsLink.

Finally, Europe PMC is making use of the EMBL-EBI Embassy Cloud to host Evidence Finder, bringing the computational aspects of the application closer to the data and providing an easy way for text mining algorithms to update outputs daily, based on incoming open access content. This mechanism is being extended to academic text mining groups that wish to routinely publish algorithm outputs to Europe PMC via the External Links service.

## SUMMARY

Europe PMC is a fast, reliable and comprehensive resource for accessing the life science literature. Based on open access and open data principles, we strive to foster a sense of ‘collaborative community’ by applying layers of value on the core full-text content through engagement with users in the many roles in which they interact with Europe PMC. Europe PMC is embedded in a big data environment, and we see smarter integration of the open full-text literature and open data as critical in supporting life science research. Europe PMC will continue to be developed toward these goals in the future.

We welcome comments and questions about any aspect of the service. The best way to contact us is via the Feedback button at the bottom of every page, or via Twitter (follow us @EuropePMC_news).

## APPENDIX

Author List:

Yuci Gou, Florian Graff, Oliver Kilian, Senay Kafkas, Jyothi Katuri, Jee-Hyub Kim, Nikos Marinos, Johanna McEntyre, Andrew Morrison, Xingjun Pi, Philip Rossiter, Francesco Talo, Vid Vartak (European Molecular Biology Laboratory, European Bioinformatics Institute (EMBL-EBI), Wellcome Trust Genome Campus, Hinxton, Cambridge CB10 1SD, UK); Lee-Ann Coleman, Craig Hawkins, Anna Kinsey, Sami Mansoor, Victoria Morris, Rob Rowbotham, (The British Library, 96 Euston Road London NW1 2DB, UK); David Chaplin, Ross MacIntyre, Yogesh Patel (Mimas, Roscoe Building, The University of Manchester, Oxford Road, Manchester M13 9PL, UK); Sophia Ananiadou, William J. Black, John McNaught, Rafal Rak and Andrew Rowley (National Centre for Text Mining, School of Computer Science, University of Manchester, 131 Princess Street, Manchester, M1 7DN, UK).
